# Stigma toward female smokers: gendered perceptions and moral judgment

**DOI:** 10.1186/s40359-026-04946-z

**Published:** 2026-06-09

**Authors:** Ibtisam Alamri, Aeshah Alamri

**Affiliations:** 1 Department of Social Studies, King Saud University, Riyadh, Kingdom of Saudi Arabia; 2 Department of Psychology, King Saud University, Riyadh, Kingdom of Saudi Arabia

**Keywords:** Female smoking, Stigma, Gender norms, Masculinity, Saudi Arabia

## Abstract

**Background:**

This study explores the gendered perceptions surrounding female smoking in Saudi Arabia, where tobacco use is shaped not only by health concerns but also by deeply rooted social norms.

**Methods:**

Employing a qualitative approach, the study draws on semi-structured interviews with 35 Saudi adults (20 women and 15 men), aged 18 and older.

**Results:**

Thematic analysis revealed a clear gendered double standard in smoking-related stigma. Women’s smoking was judged more harshly than men’s and was often interpreted as a violation of femininity, modesty, moral propriety, and family reputation. Participants also linked female smoking to poor upbringing, emotional instability, and failure to meet expected reproductive or caregiving roles. In contrast, men’s smoking was largely normalised, and at times associated with masculinity, despite its recognised health risks. Viewed through gender role, patriarchal, and social control perspectives, these findings show how smoking stigma functions not only as health-related disapproval but also as a mechanism for regulating women’s behaviour and reinforcing gendered expectations.

**Conclusions:**

The findings show that smoking is a socially regulated practice in Saudi Arabia, particularly for women. Tobacco control strategies should therefore be gender-sensitive, non-moralising, and culturally informed. Public health efforts should frame smoking as a health issue, avoid reinforcing gender stereotypes, and provide confidential cessation support, especially for women who may fear social judgment. Addressing these gendered double standards is important for reducing stigma and promoting more equitable health outcomes.

**Supplementary Information:**

The online version contains supplementary material available at 10.1186/s40359-026-04946-z.

## Social stigma toward female smokers: gendered perceptions and moral judgment

Smoking remains a major global public health challenge, contributing to millions of preventable deaths, substantial economic losses, and widening health inequities. According to the World Health Organisation [1], approximately 1.2 billion people use tobacco worldwide, causing nearly 8 million deaths annually, including 1.3 million deaths among non-smokers exposed to secondhand smoke. Although global tobacco use has declined over the past two decades, the emergence of new nicotine products and continued tobacco industry marketing sustain the epidemic [2]. Beyond its physical health consequences, smoking is also associated with significant social and psychological burdens, including loneliness, reduced social contact, lower community engagement, stress, depressed mood, anxiety, low self-esteem, and diminished quality of life [3–9]. These effects extend beyond individual well-being, influencing how smokers are perceived and treated within society, which has increasingly been conceptualised as smoking-related stigma.

### Stigma of smokers

Tobacco use has long carried social stigma, which can be referred to as the social disapproval and negative stereotyping directed at smokers, marking them as having a tainted or morally questionable status in society [10]. Smoking has been denormalised in public health discourse, reducing smoking rates but also contributing to smoking stigma [10, 11]. Men continue to smoke at higher rates than women and generally experience less stigma. In contrast, smoking among women varies across cultural contexts and is often more socially condemned. In many low- and middle-income countries, strong social and religious norms make female smokers more visible and subject to harsher judgment. Even in high-income countries where female smoking is more common, women still face greater stigma than men [12–15].

Gendered perceptions of smoking reflect broader social expectations about femininity and masculinity. Women’s smoking is often viewed as less acceptable and as a violation of feminine norms, whereas men’s smoking is more frequently normalised or associated with independence, masculinity, and stress relief [11]. For example, Thoma and Weber [16] found that smoking increased the perceived attractiveness and masculinity of male models but reduced the attractiveness of female models. Although stigma may discourage smoking, excessive stigma can produce harmful psychological and social consequences, especially for women, including internalised stigma, depression, anxiety, low self-esteem, emotional distress, social isolation, damaged relationships, discrimination, and reluctance to seek cessation or healthcare support due to fear of judgment [10, 11, 14, 17–22].

### Drivers of stigma toward female smoking

The literature identifies several factors that contribute to the stigma surrounding female smokers, with gender norms being among the most central. In many societies, smoking is viewed as inconsistent with traditional ideals of femininity, particularly because it has historically been associated with toughness, independence, and rebellion, traits that are often more socially acceptable for men than women [18, 23]. In Confucian-influenced South Korean culture, for example, women are traditionally expected to be self-sacrificing and family-oriented, making smoking appear incompatible with feminine ideals [13]. Similarly, in traditional or religious societies, women’s smoking may be perceived as a violation of expectations related to modesty, purity, and appropriate feminine behaviour, leading to stronger social condemnation [14]. This stigma may be intensified where female smoking is relatively uncommon, as women who smoke become more visible and are perceived as violating both health and gender norms, particularly in rural or conservative communities [24, 25].

Another important driver of stigma is the social ideal of motherhood and the expectation that women, especially mothers, prioritise the well-being of their children. Because smoking is associated with serious health risks, women who smoke during pregnancy or while raising children are often judged more harshly for failing to meet standards of maternal responsibility [14, 26]. As a result, many smoking mothers report guilt, fear of judgment, and efforts to protect their identity as responsible mothers, such as avoiding smoking in public or comparing themselves to heavier smokers to reduce internal conflict and social disapproval [18, 21]. Media and tobacco marketing also contribute to this tension by portraying female smokers as glamorous, rebellious, or empowered, while many communities continue to judge women’s smoking negatively in everyday contexts such as families, workplaces, and pregnancy [11, 14, 23, 27].

### Current study

Smoking remains a culturally sensitive issue in Saudi Arabia, particularly for women. Although the overall prevalence of smoking in Saudi society is considerable, the rate among females has historically been much lower than that of males. For example, a national survey of individuals aged 15 and older in Saudi Arabia estimated that approximately 26–27% of men smoke, compared to just 2% of women [28]. However, researchers have noted that these figures are likely conservative, as the study did not include other forms of tobacco use, such as waterpipe (shisha), electronic cigarettes, and smokeless tobacco (e.g., chewing tobacco). Other studies have found that gender differences in tobacco use vary depending on the product. While cigarette smoking remains predominantly male, waterpipe and certain non-cigarette forms of tobacco show relatively higher use among females in specific subpopulations. Additionally, electronic cigarette use is rising among the young population, particularly in university settings [29–31].

Research suggests that the recent gradual increase in smoking among women is linked to overlapping social and cultural shifts. In Saudi Arabia, women’s expanding participation in education, employment, and public life, together with broader societal transformation, may have gradually loosened some traditional constraints on female behaviour, creating greater opportunities for experimentation with smoking. This social change has contributed to the erosion of longstanding taboos surrounding female smoking and has led to a perception among certain younger and urban populations that it is becoming more socially acceptable [32–34]. Many young females view tobacco use, especially waterpipe, as fashionable and relaxing, which lowers perceived risk and encourages more frequent use [32, 35]. For some, smoking, particularly in private or female-only gatherings, is described as a means of coping with stress, family expectations, and academic or professional pressures. This coping function has become increasingly relevant as women take on new roles in the midst of rapid social change [32, 33].

Previous literature has established that women face unique stigmatisation as smokers and has identified several contributing factors. This issue is especially evident in conservative contexts, where women’s smoking is often viewed not only as a health risk but also as a moral or social violation [13–15]. In Saudi Arabia, where gender roles are strongly shaped by traditional, religious, and tribal values, women who smoke may therefore be subject to especially harsh judgment. Despite the growing visibility of female smoking, however, the social and psychological consequences of smoking-related stigma for Saudi women remain poorly understood. Existing studies have largely focused on health-related dimensions, such as prevalence, motivations, and desire to quit, leaving limited understanding of how stigma is constructed, experienced, and justified in this cultural context. This gap is important because smoking-related stigma can harm women’s mental health and social relationships, discourage help-seeking for cessation support, and ultimately undermine tobacco control efforts. Accordingly, the present study addresses two key questions: (1) How is smoking-related stigma gendered in Saudi Arabia? and (2) What social and psychological mechanisms explain how this stigma operates?

## Methods

### Study design

This study employed a qualitative exploratory design to investigate gendered perceptions of stigma toward female smokers in Saudi Arabia. Qualitative methods are particularly suited to exploring socially sensitive topics as they allow for an in-depth understanding of participants' attitudes, justifications, and lived experiences. Data were collected through semi-structured interviews, and the resulting transcripts were analysed using thematic analysis following the six-phase approach of Braun and Clarke [36]. This design enabled the researchers to capture diverse perspectives from both men and women, while remaining attentive to the cultural and moral nuances of smoking stigma.

### Participants and recruitment

After receiving ethical approval for the study from the Research Ethics Committee at King Saud University, the researchers recruited participants for this study through their personal and professional networks in Saudi Arabia. These networks included colleagues, university contacts, and people from the local community. The sample included both women and men, and both smokers and non-smokers, to capture different points of view. Family members and close friends were not included in order to reduce bias and keep a professional distance between the researchers and the participants. The aim of the study was not to obtain a statistically representative sample, but to collect rich qualitative data that could help to understand how people think about and describe women who smoke.

The researchers then contacted the potential participants via text messages, emails, and phone calls, and were given a clear explanation of the aim of the study and what participation would involve. People were invited to take part, men and women, if they were aged 18 years or older, of Saudi Arabian nationality, and willing to share their views about women’s smoking and social attitudes towards it. Participation was entirely voluntary, and all participants were informed about their right to withdraw from the study at any time without any negative consequences. Verbal or written informed consent was obtained from each participant before the interviews began.

The final sample consisted of 35 participants (20 women and 15 men) aged between 18 and 58 years. The sample included both smokers and non-smokers. Most participants identified as non-smokers, while seven (one of them was a woman) were current smokers and one was a former smoker. The aim was not to conduct a statistical comparison between men and women, but rather to gather diverse gendered perspectives on the stigma surrounding women’s smoking. Demographic characteristics of the sample are presented in Table 1.

**Table 1:** Demographic characteristics of the sample

Variable	Category	n	%
Sex	Female	20	57.1
	Male	15	42.9
Age	18–24	8	22.9
	25–34	12	34.3
	35–44	8	22.9
	45 and above	7	20.0
Smoking status	Non-smoker	27	77.1
	Current smoker	7	20.0
	Former smoker	1	2.9

### Data collection

Data were collected through qualitative semi-structured interviews with Saudi men and women. The interview guide was developed based on a literature review of gendered smoking stigma, focusing on five domains: perceptions of female smokers, comparison to male smokers, social consequences, justifications, and future change. Open-ended questions and probes were drafted, then pilot-tested with three Saudi adults; minor wording adjustments were made to ensure cultural appropriateness and neutrality. The final guide was developed specifically for this study and has not been published elsewhere. An English version is provided as a Supplementary File. Example questions included: "When you hear the phrase 'a woman who smokes', what is the first thing that comes to your mind?" and "How would you describe society's view of a woman who smokes?" Probing questions were used when needed to encourage more detailed answers and examples.

All interviews were carried out by two Saudi female researchers who share the same language, religion, and broader cultural background as the participants. Being insiders to this social context made it easier to approach potential participants, build rapport, and discuss a topic such as women’s smoking. Conducting the interviews themselves and later coding and writing up the data helped the researchers to understand references to issues such as reputation, honour, and appropriate gendered behaviour in the way participants intended them. At the same time, the researchers recognised that their shared background and personal views might influence what participants chose to disclose and how the accounts were interpreted. To address this, they discussed their field notes together, compared their initial codes and themes, and reached agreement on the final interpretations to strengthen the credibility and trustworthiness of the findings.

Interviews were held face to face, by telephone, or via Zoom, depending on each participant’s preference and availability. For in-person interviews, participants chose the location, which included cafés and workplaces. All interviews were conducted in Arabic and typically lasted between 30 and 64 min. All interviews were audio-recorded with participants’ permission and then transferred to a secure, password-protected drive to which only the researchers had access. Each participant was assigned a pseudonym, and any identifying details were removed from the transcripts to ensure anonymity; all names reported in the findings are therefore pseudonyms. Data saturation was reached around the 27th interview, when no new codes, themes, or insights emerged, and responses became repetitive. Additional interviews were conducted to confirm the stability of the identified themes. All interviews were manually transcribed in Arabic in Microsoft Word shortly after each session to reduce the risk of data loss and to keep the content fresh in the researchers' minds. All analysis, including coding and theme development, was carried out in Arabic to preserve the cultural and linguistic nuances of participants' accounts. Only selected quotes were translated into English during the writing process; these were translated by the first author and independently back-translated by a bilingual colleague to ensure accuracy and consistency with the original meaning.

### Data analysis

To analyse the data, the researchers used thematic analysis as described by Braun and Clarke [36]. The analysis took place in several steps. First, after transcribing all of the interviews, they were carefully read several times to gain a good overall understanding of the data. Second, the researchers coded the transcripts line by line, using short labels to mark pieces of text that were relevant to the research question (for example, comments about gender, stigma, or social judgment). Third, similar codes were grouped to form initial themes by looking for links and patterns between them. These themes were then reviewed and refined by checking them against the coded extracts and the full data set, to make sure they accurately reflected what participants had said. After that, each theme was given a clear name and definition that captured its main idea.

The final thematic structure comprised three interconnected themes: Theme 1: gendered stigma of women's smoking: moral, cultural, and social boundaries; Theme 2: men's smoking as normal: masculinity and social acceptance; and Theme 3: justifying the double standard: body, marriageability, motherhood, and reputation. Throughout this process, the researchers explicitly examined how these themes interconnected, paying particular attention to the contrast between the first theme, the stigmatisation of women's smoking, and the second theme, the normalisation of men's smoking, as well as the justifications participants offered for this asymmetry, which is the third theme. Finally, the themes were written up in the results section, with illustrative quotes used to show how participants expressed each theme.

### Findings

All participants acknowledged that women who smoke do exist, even if they are rarely seen smoking in public. They explained that, unlike men, women often hide their smoking from others, especially from their families. Nouf, a female participant, explained: "*Yes, there are women who smoke, and in my opinion, the number of girls has increased lately, but many of them still smoke in secret because they are afraid of how people will see them*." Khadija, another female participant, commented: "*She feels ashamed to smoke in front of people and in society, and I expect that a woman who smokes in our society does so away from her family*". Women’s smoking emerged as a socially regulated one. Participants described secrecy as a response to anticipated judgment and to the moral and cultural meanings attached to women’s smoking in Saudi society.

### Gendered stigma of women’s smoking: moral, cultural, and social boundaries

This theme shows how participants framed women’s smoking as a gendered stigma. Accounts moved from viewing it as a loss of femininity to describing it as unfamiliar and culturally unacceptable, often extending into judgements about respectability. Participants also linked it to religious–moral violation, to psychological or social problems, and finally to blame directed at the woman’s family and upbringing.

### Stigma as loss of femininity

This subtheme was the most frequently expressed form of stigma across the interviews. The majority of participants described women’s smoking as incompatible with femininity. They stated that it is seen as a violation of feminine norms and as conflicting with how women are expected to look and behave in Saudi society. Abdullah, a male participant, confirmed this point:

*Honestly, I don’t like this sight at all. I feel she loses something of her femininity when she’s holding a cigarette. Maybe I smoke myself, but I still feel that the way a girl looks when she smokes doesn’t suit her at all.*

When asked to elaborate, he added: "*A cigarette in a girl’s hand ruins her image, and it makes her smell like smoke. A girl is supposed to smell like perfume and look soft and delicate, and smoking is the complete opposite*". As Abdullah focused on how smoking affects a woman’s feminine look and scent, other participants went further and linked smoking to reduced attractiveness in men’s eyes. Omar, a male participant, said:

*I think a woman becomes less feminine when she smokes; her attractiveness decreases. When a man sees a woman smoking, she falls in his eyes, and he may feel disgust. A woman’s femininity is associated with caring about herself, her appearance, and her style, so when she smokes, she breaks femininity.*

Beyond appearance and desirability, a few accounts described femininity in social and moral terms, linking it to modesty and appropriate public behaviour. Fatimah, a female participant, described women’s smoking as shameful and inappropriate, explaining that because "*we associate her image with modesty, calmness, and softness, so if she smokes, it is as though she has stepped outside the boundaries they have set for her*''. Ahmed, a male participant, supported the same expectation by describing the *proper* feminine image as modest, calm, and well-behaved, adding that smoking is widely treated as a male-coded practice:

*We see a girl as someone who should be modest, calm, conservative, and well-behaved. So when she does something like this, it feels like she is going against what we are used to; smoking is seen as something for men only.*

In this sense, modesty operated as a boundary of femininity, so smoking was framed as crossing that boundary rather than as a neutral personal habit.

Building on these, femininity was defined not only by appearance but also by modest conduct; therefore, a woman who smokes could be read as losing femininity and moving closer to a *masculine* style of behaviour. Feryal, a female participant, stated:

*Smoking is the enemy of femininity. A woman is known for her femininity, attractiveness, and softness, so the moment she engages in this bad habit, one that makes her resemble men, she inevitably loses something of her femininity.*

Tala, another female participant, built on this idea by linking women’s smoking to imitating men and to stepping outside expected feminine behaviour in public. She explained: "*She is trying to imitate men because smoking is for men*". She then connected this to the social image of femininity, where women are expected to appear soft and calm and to maintain a *proper* public presence:* "We expect a woman to be soft and calm, and for her behaviour to suit that image. But smoking in public doesn’t express that you’re a ‘feminine’ woman. As for the smell, women usually care a lot about their scent and cleanliness*." Across these accounts, women who smoke were perceived as violating core ideals of femininity across multiple dimensions: physical appearance and scent, sexual desirability, modesty, and the avoidance of male-coded behaviours. This formed the most frequent and fundamental form of stigma directed at female smokers.

### Stigma as unfamiliarity and unacceptability

The participants often described women’s smoking as *strange* because it is rarely seen in public and does not fit with social expectations of how women should behave. Mishal, a male participant, explained:

*Honestly, the first thing that comes to my mind is that it’s a bit strange, because we don’t usually see girls smoking around us. It feels like something new or different, and it’s not always accepted*.

Khadija, a female participant, supported this point directly by linking *strangeness* to rarity: "*Because it’s something strange and very rare in society when it comes to women*. "

Beyond rarity, women’s smoking was often linked to gendered expectations about how women should behave. Omar, a male participant, explained:

*To be honest, the first feeling I get is surprise, because I’m not used to seeing women smoke. I feel it’s a habit that doesn’t match the image of a woman that we have in mind. Even if she is free to do what she wants, it still feels somewhat striking.*

This *image of a woman* was often linked to idealised motherhood. Mohammad, a male participant, noted: *"Smoking for a woman is something we find strange, especially because a woman may be a mother, and the view of a mother is often idealised"*.

Participants also viewed women's smoking as culturally unacceptable. Abdulrahman, a male participant, linked this to breaking traditions: *''They see her as someone who has broken traditions or doesn’t care about what society says…".* He further explained that smoking by women was seen as inconsistent with accepted cultural values*: "as a society, we reject these habits, we weren’t raised on them, and our culture doesn’t accept this kind of thing.* Hadeel, a female participant, expressed a similar view and added that rejection is also tied to appearance and public presentation. She explained that society rejects these practices because "*it doesn’t fit, neither outwardly nor in appearance, nor does it fit with customs and traditions*."

Additionally, cultural rejection quickly became a judgment about respectability, as Amal, a female participant, explained:

*Society believes that a woman must be respectful of our customs and traditions, so when they see her smoking, they will definitely disapprove and criticise her, and her reputation becomes damaged because she is seen as not respectable.*

This judgment was also expressed through the language of rebellion. Sami, a male participant, stated: "*She is a woman who rebels, and she is seen as undesirable in any kind of relationship, whether as a colleague, a wife, or even as a daughter among relatives".* He further added that* "It is considered shameful for someone to say that they have a woman in their life who smokes".* Sama, a female participant, shared a similar view and added that this judgment can extend to family relations, where women may be described as disobedient to their parents: "*People view her negatively and see her as rebellious against her society and family, because they think she may be disobedient to her parents*".

Overall, women who smoke were stigmatised as both unfamiliar, rare, strange, surprising, and culturally unacceptable, contrary to customs, damaging to reputation, associated with rebellion and family disobedience. These forms of stigma reinforced each other: rarity made the behaviour seem strange, and cultural norms labelled it as illegitimate.

### Stigma as moral, religious, psychological, and social deviance

Many participants viewed women’s smoking as a violation of religious and moral values, speaking about this judgment as if it were a widely accepted social norm. Fatimah, a female participant, reflected this:

*People see it in a negative way, and that’s natural because women’s smoking is seen as religiously forbidden and morally rejected in our society. Many see her as someone who doesn’t respect her religion.*

Building on this perspective, some participants went further by describing women’s smoking as a sign of weak religious commitment. Nora, a female participant, stated: "*I see her as weak in her faith and careless, and as someone who does not take anyone into consideration, not her faith, nor her God*"*.* In a few accounts, the stigma escalated further as Khalid, a male participant, used highly critical language, describing women who smoke as a source of corruption: "*She is a woman who is abnormal, low, Immoral, and a source of corruption… she most likely ended up in this situation because she is morally corrupted."*

Alongside religious and moral condemnation, some participants interpreted women’s smoking through a problem lens. For example, Abdulaziz, a male participant, suggested: "*A woman who smokes must have social or psychological problems*". Other participants offered a less harsh but related explanation, Hadeel, a female participant, said: "*Women smoke because of psychological pressure and family problems… they try to escape from it and turn to smoking, even though it is not a solution*." Similerly, Ahmed, a male participant, said: "*I would wonder what led her to this, maybe pressure or stress… I know that smoking often starts because of pressure or someone’s influence*."Jameelah, a female participant, developed this point by describing smoking as a way to release worries and suppressed emotions when other coping options feel limited, she said:

*She is someone who is under pressure, has worries, suppressed feelings, and difficulty focusing. She releases these negative emotions and energy through smoking, because she may not have other ways to cope or express what she is feeling.*

A further variation focused on motivation and self-presentation as Abdulrahman, a male participant, linked women’s smoking to weakness and limitation: "*It can be a sign of a weak personality… trying to show strength or courage… or to attract attentio*n." This interpretation also appeared in accounts that framed smoking as competing with men and copying what men do. Khalid, a male participant, explained that some women may smoke to prove they are *just as strong*. He said:

*Some girls compete with men and try to prove that they are just as strong. They say, ‘What is something I wasn’t allowed to do before? So, they start working, becoming employees, and driving. Then they ask, ‘What else do men do? smoking. So, they say, ‘I’ll smoke too.*

Collectively, female smokers were stigmatised across multiple, intersecting levels: religiously as sinful or weak in faith, morally as shameful or corrupt, and psychologically as stressed, weak, or imitating men. These layers of stigma reinforced one another, shaping the female smoker as socially deviant rather than as someone responding to broader social pressures.

### Stigma as family failure

The stigma was not limited to the woman herself but also extended to her family. Women’s smoking was often viewed as a sign of poor upbringing and insufficient parental guidance, suggesting that the family had failed to provide proper direction. Malak, for example, a female participant, stated: "*The first thing that comes to my mind is that she is ignorant, because there is no one guiding her, even though she knows the harms of smoking for health and for herself*". This judgment was sometimes reduced to blunt everyday labels that directly tied smoking to manners and upbringing. Tala, a female participant, stated: "*She is ill-mannered (not properly brought up)*."

Some participants developed this judgement further by widening *poor upbringing* into a broader conservative moral framework and by describing harms that extend beyond the woman to the household. Mohsen, a male participant, explained:

*She is not properly brought up according to the norms of our conservative society, and she does not care about her health, causing problems for herself, her family, and any children she may have.*

Ali, a male participant, combined this upbringing narrative with a coping explanation, suggesting that smoking reflects both *poor upbringing* and an attempt to escape pressure: "*It reflects poor upbringing and shows that she was not raised properly, since she has used smoking as a way to escape from the pressures of life*."

Broadly, participants extended the stigma from the individual woman to her family, interpreting her smoking as evidence of failed upbringing and parental neglect, while also acknowledging broader social pressures that may drive women to smoke.

### Men’s smoking as normal: masculinity and social acceptance

Several participants highlighted that such judgements are gendered. Hessa, a female participant, summarised this contrast by noting that men’s smoking is more readily accepted: "*The difference is very clear: smoking is accepted for men, but a woman who smokes is seen as a kind of rebellion or a violation of customs*"*.* Similarly, Muhammad, a male participant, added: "*The same act (smoking) is seen as a personal freedom or a bad habit for men, but as shameful behaviour and scandal for women*." Sama, a female participant, expressed this very clearly and observed that "*A man who smokes is considered normal; people might not even question it. In contrast, a woman who smokes is often met with shock and becomes the subject of gossip*."

Across the interviews, the majority of participants described men’s smoking as an unhealthy but socially familiar and ordinary practice. For example, Saud, a male participant, said:

*Smoking for men is unhealthy but generally seen as completely normal; it has become socially ordinary and may even sometimes be viewed as a ‘masculine’ habit. No one criticises him or links smoking to his morals the way we do with women.*

Another male participant linked this acceptance to how widespread smoking is among men, Abdulaziz said: "*At present, there is some degree of acceptance because some men are afflicted with it, but overall, it is acceptable*."

Alongside seeing men’s smoking as a normal and familiar behaviour, several participants also noted that some people in society link smoking with masculinity, strength, and toughness. Amal, a female participant, mentioned that "*Some people see smoking in men as part of being masculine and tough, and they think it is normal for a man to smoke*''*.* Khalied, a male participant, confirmed Amal’s view and suggested that this association has shifted over time, particularly among younger men, he said:

*In the past, it was seen very negatively, as something lacking in dignity, but now this view has changed, and people see it as a sign of manliness and as a form of showing off, especially among young people and adolescents*.

However, a few participants noted that this normalisation is not absolute. Sami, a male participant, explained that although men’s smoking is judged less harshly than women’s, it can be rejected in certain contexts, especially marriage: "*Many religious and conservative families refuse to marry their daughters to a man who smokes''.*

Overall, these accounts show how men’s smoking is widely normalised and sometimes masculinised, while women’s smoking attracts stronger moral and social stigma.

### Justifying the double standard: body, marriageability, motherhood, reputation

Across the interviews, the participants recognised a clear double standard in how smoking is judged for men and women, and offered explanations that helped legitimise it. These accounts draw on concerns about the female body and health, marriage and social relationships, motherhood, and women’s reputation.

### Health and the female body

Many participants believed that smoking affects the health of both men and women, but that its impact on women is far greater than on men. Mohsen, a male participant, stated: "*Men are physically stronger than women, in terms of body structure and nature*", but then went on to emphasise that the main difference, in his view, lies in the potential harm to pregnancy and the fetus. He explained:

*I see that a man can smoke since the health risks are limited and confined to him, whereas a woman may be pregnant and harm her fetus, so the health risks are not limited to her alone. It is true that a man can also harm his family, but not in the same way as a woman harming her unborn child.*

Mohsen’s view was supported by Hadeel, a female participant, who believes the nature of a man’s body and his ability to endure are different from a woman’s "*A woman has pregnancy, childbirth, and childcare, and her body is weaker than a man’s, so the impact on her is stronger."*

Additionally, some participants highlighted another health-related reason that differentiates the impact of smoking on women, namely its effect on physical appearance. They suggested that beauty and good appearance matter more for women than for men. Jameelah, a female participant, explained:

*In the case of a woman who smokes, her facial features and overall health will change. For example, her lips may turn dark, so she might feel the need to undergo cosmetic procedures. Signs of fatigue and paleness will also appear on her face, which will reduce her attractiveness.*

Dalal, another female participant who smokes, supported Jameelah’s view and added *hormone issues* as another factor. She said: *''Smoking ruins the skin and makes it look dull, and it affects the body. It impacts fertility, pregnancy, childbirth, and delivery."* She added that *"It also changes the voice, causes a constant cough, bad breath, and darkened lips. It also affects women's internal health and disrupts their hormones".*

Overall, participants justified stricter judgment of female smokers by claiming that smoking poses greater physical risks to women, particularly regarding reproduction and appearance.

### Relationships and marriage

Socially, the participants offered several reasons to explain what they saw as an inequality between men and women, emphasising that smoking often undermines a woman’s social relationships. Sama, a female participant, felt that being a smoker might *isolate her from society*. She explained:

*Smoking may influence how people treat her, and they may not accept her as a smoker as long as society rejects the idea in the first place. People may distance themselves from her, which can lead to social isolation, and she may be described as socially deviant and disrespectful.*

Participants also talked about marriage as another area where this inequality becomes very clear. They believed that the chances of a man who smokes getting married are much higher than those of a woman who smokes. Abdullah, a male participant who smokes, explained that: "*Maybe in terms of health only, but not in terms of how people see you. I’m a smoker, for example, and no one has ever said anything to me or criticised me. Society is more lenient with men who smoke.* "However, he stressed that this would not be the case for a woman who smokes:

*In marriage, no one will propose to her [woman]. People will talk about her in a negative way, and her female colleagues and friends will distance themselves from her. Smoking, for a girl, is seen as a very negative trait, even if she is respectable.*

Similarly, Salem, a male participant, reinforced Abdullah’s view, "*Of course, no man would accept a woman who smokes, and no workplace management would accept a woman who smokes in its offices”*. Thus, female smokers were seen as socially and maritally undesirable, whereas male smokers faced no such consequences.

### Motherhood and caregiving

Besides the accounts about social isolation and marriage, several participants linked society’s rejection of women who smoke to their role as mothers and caregivers. They believed that this role requires women to stay away from *bad* habits such as smoking, because they are seen as role models for children. Munirah, a female participant, explained this view:

*She was created to be a mother, a sister, and a daughter, and to belong to the home to raise a righteous generation. She was created to raise her children in the best way, and to be a soft and beautiful woman, not for these things [smoking].*

Similarly, Khalid, a male participant, indicated that this caregiving role makes a woman’s impact on the family stronger than a man’s: "*She influences her children, her husband, and her society; this means that her negative impact on the family is greater than that of a man. "* When he was asked whether a man who smokes can also negatively affect his family and children, he replied that it is still not the same as with a woman:

*But it is not the same as with a woman. A woman has a very strong influence, especially on her children: in how they think, in what is seen as normal or not normal. The mother’s influence is always greater than the father’s in raising and educating children.*

Sami, another male participant, reinforced this view by stressing the woman’s role in educating and raising children. He described the mother as a *teacher* and a *caregiver* and linked this to greater social sensitivity towards women’s engagement in what society considers bad habits:

*The mother is a teacher and a caregiver, so a woman’s engagement in what our society considers bad habits is seen as more sensitive. Our society is less willing to accept these habits from women than from men, whether it is smoking or other behaviours.*

Overall, participants constructed women's smoking as incompatible with their roles as primary caregivers and moral exemplars, arguing that a mother's influence on children is unique and therefore her moral conduct is subject to stricter scrutiny.

### Reputation and morality

Ruining a woman’s reputation was another reason participants gave to justify the inequality between men and women who smoke. Several participants believed that women who smoke are automatically seen as having a bad reputation. Khalid, a male participant, stated that "*It is already wrong in the first place, and girls cannot easily get cigarettes in ‘closed’ places*." When he was asked what he meant by "*closed places*", he clarified:

*I mean a careful, health-conscious family that watches over its children…. so it is usually assumed that something negative is going on, like that someone ‘bad’ must have given them to her or that she has fallen in with the wrong kind of friends.*

A similar view was shared by Sami, a male participant, who stressed that a man who smokes is not usually considered an abnormal person and that his only *flaw* is that he smokes. However, he believed that: "*smoking in the case of women is often linked to many other bad things about her. For a woman, it comes as a whole negative package*".

The participants also referred to a folk proverb, "*al-rajul shayel ʿeebah*", which they used to justify why a man’s reputation is less likely to be damaged. A close translation of this proverb is *"a man carries his own flaws*." Saud, a male participant, drew on this idea and explained: "*A man has more social space, and his mistakes are easily forgiven in our society, whereas any behaviour by a woman is blown out of proportion*". The idea behind this proverb was also reflected in the account of Abdulrahman, a male participant, who described how people respond differently to men and women who smoke:

*A man’s flaws are seen as less serious….. He might be generous, and they overlook his flaw. But for a woman, no, it’s considered a kind of moral corruption. Even if she has good character, her smoking is still seen as wrong.*

Collectively, Participants viewed smoking as automatically damaging a woman's reputation and signaling broader moral failings, while men's smoking was viewed as an isolated, forgivable flaw.

## Discussion

This study examined the social meanings attached to smoking in the Saudi context, with particular attention to how gender shapes smoking-related stigma. Although smoking has been widely studied as a public health concern, less attention has been given to how social norms and gender expectations shape stigma and everyday responses to smoking in Saudi Arabia. Drawing on participants’ accounts, the findings reveal a clear gendered double standard; women who smoke were subject to intense moral, social, and cultural condemnation, whereas men’s smoking was largely normalised despite being recognised as unhealthy. This disparity is consistent with research from other societies showing that female smokers often face stronger negative stereotypes and social sanctions than male smokers [11, 13, 15, 16, 37].

To make sense of these gendered patterns, the present discussion draws on four complementary theoretical perspectives. Gender role theory explains how societies create different expectations for men and women, and why women who depart from accepted feminine roles, such as by smoking, may face social backlash [38–40]. Patriarchal theory adds another layer by showing how the enforcement of these norms can help maintain gendered power relations, where women’s autonomy is more closely monitored and judged than men’s [41, 42]. Social control theory further explains how stigma works as a form of social regulation, through which communities sanction behaviours seen as threatening to family reputation, social order, or shared moral values [43, 44]. Finally, Goffman’s [45] theory of stigma helps explain the personal and social experience of being marked by a “spoiled identity”, including concealment, shame, and the management of discrediting information. Together, these perspectives suggest that in the Saudi context, smoking-related stigma is intensified when women’s behaviour is perceived as violating expectations of femininity, morality, and family respectability.

Consistent with gender role theory, the stigmatisation observed in this study reflects broader patterns documented in previous research. Across many cultural contexts, women’s smoking is constructed as a violation of traditional femininity and is often viewed as incompatible with ideals of modesty, respectability, and appropriate womanhood [11, 13, 16, 46]. In the present study, participants described female smokers as having “lost their femininity” or as behaving immodestly. Women’s smoking was also portrayed as strange, surprising, and culturally unacceptable. These responses can be understood as backlash against women who violate strongly prescribed feminine norms, where smoking is judged not only as unhealthy or deviant but also as gender-incongruent [39, 40]. This reaction is particularly understandable in the Saudi context, where public smoking among women has long been taboo and remains relatively uncommon. As a result, women who smoke publicly may attract heightened scrutiny because their behaviour is perceived as both socially transgressive and inconsistent with accepted ideals of womanhood [14, 15, 23, 25].

Despite these cross-cultural similarities, our findings also reveal context-specific differences in how gendered smoking stigma is expressed in Saudi Arabia. One notable feature was the explicit use of religious and moral language when condemning women’s smoking. Although moral disapproval of female smokers has been reported in other cultural contexts (e.g., [14, 15], participants in the present study often expressed this disapproval through religious terms, such as describing smoking as “sinful”. This reflects how religious and moral values can become part of the social language through which gender expectations are interpreted and justified. In contrast, studies from some Western contexts tend to emphasise health risks or social inconvenience, such as secondhand smoke or smell, as reasons for disapproval [10, 37]. From a social control perspective, this suggests that stigma operates through culturally specific forms of moral regulation, sanctioning behaviours perceived as threatening to family reputation, social order, or shared values [43, 44]. In the Saudi context, this regulation appears to be expressed not only through health concerns, but also through moral and religious meanings attached to women’s behaviour.

Beyond religious and moral disapproval, participants also stigmatised female smokers by attributing their behaviour to psychological problems, weak self-control, or poor upbringing. These explanations framed women’s smoking not simply as a health-related behaviour but as evidence of personal or familial deficiency. Goffman’s [45] concept of stigma helps explain this process, as female smokers were marked by what he described as a discrediting “blemish of individual character”. Such framing shifts attention away from the social and cultural pressures that may shape smoking and places responsibility on the individual woman. Patriarchal theory adds that these judgments also help maintain gendered double standards by holding women to stricter moral and behavioural expectations than men, and by treating women’s deviation from these expectations as a sign of deeper personal or family failure [37, 41, 42, 47].

In contrast to women’s smoking, men’s smoking was widely perceived as normal, even when participants recognised it as unhealthy. This gendered contrast is well documented in prior research, which shows that men generally use tobacco at higher rates and face less stigma than women [10, 18, 48]. In the present study, some participants noted that although they personally disliked smoking, they still viewed it as ordinary for men and often socially unremarkable. Others associated men’s smoking with confidence, masculinity, or “being manly,” echoing prior research and cultural representations of cigarettes as symbols of toughness, independence, stress relief, and coolness. [23, 49, 50]. In line with gender role theory, this asymmetry can be understood through gender congruence: smoking is more easily tolerated among men because it aligns with culturally familiar masculine traits such as autonomy, risk-taking, and toughness [38]. Patriarchal theory adds that men’s smoking is often treated as a personal choice, whereas women’s smoking is interpreted as a matter of family reputation and social morality [41, 42]. Thus, smoking may be condemned as unhealthy yet still tolerated, or even partly normalised, among men, while being framed as a serious moral and social failing among women.

A key contribution of this study lies in examining the specific rationales participants used to justify unequal judgments of female and male smokers. One common justification is centred on women’s bodies and perceived health responsibilities. Many participants argued that smoking is especially harmful for women because of their reproductive capacity, reflecting a protectionist view of women as potential mothers. Previous research shows that women’s smoking, particularly during pregnancy, is often stigmatised as a failure of maternal responsibility [51, 52]. In the present study, however, this concern extended beyond pregnancy as simply being a woman was treated as carrying future reproductive responsibility, which intensified disapproval of smoking. Participants also described women’s bodies as more biologically vulnerable to smoking-related harm, citing risks such as hormonal imbalance, infertility, and accelerated ageing. Although some health risks are gender-specific, these claims often went beyond medical concern and reinforced broader expectations that women should preserve bodily health, attractiveness, and reproductive capacity [19]. Social control theory suggests that when women’s bodies are treated as matters of family and social concern, the regulation of women’s smoking can be presented as protection rather than judgment [43, 44]. Patriarchal theory adds that such reasoning may also reflect wider gendered power relations, in which women’s reproductive and bodily choices are more closely monitored than men’s [42].

Another major justification for the unequal treatment of female smokers is centred on women’s expected roles as wives and mothers, the very roles that gender role theory identifies as the prescriptive core of traditional femininity [38], and that social control theory identifies as the institutional sites through which family stability and cultural continuity are reproduced [44]. Participants frequently suggested that a woman who smokes would fail as a nurturer and moral example within the family. This aligns with prior research showing that mothers who smoke often face intensified stigma because they are seen not only as harming their own health but also as violating caregiving responsibilities [14, 53]. In the present study, however, this logic extended beyond actual motherhood, as participants often treated all women as future mothers. According to gender role theory, this shows how expectations linked to motherhood can be applied to women even before they become mothers, making smoking appear incompatible with accepted ideals of femininity and family responsibility [38]. By contrast, fathers who smoke were rarely judged as harshly, and some participants noted that even conservative families might overlook men’s smoking altogether, further illustrating the disproportionate moral scrutiny directed at women [18].

In some cases, this judgment also extended to the woman’s family. Some participants suggested that a woman who smokes must come from a “bad family”, reinforcing the idea that her behaviour reflects poorly on her parents and their values [27]. This form of collective blame is especially important in contexts where women’s behaviour is closely linked to family reputation and social standing. From a patriarchal perspective, such judgments place responsibility not only on the woman herself but also on her family, encouraging parents, siblings, husbands, and extended relatives to monitor women’s behaviour in order to protect collective honour [54, 55]. Social control theory similarly helps explain why family-based sanctions can be powerful: they are informal, continuous, and embedded in everyday relationships [43]. Thus, women’s smoking is judged not simply as an individual habit, but as a perceived failure of femininity, family responsibility, and social upbringing.

One striking finding was that participants believed women often conceal their smoking because of fear of social judgment, stigma, and harm to their personal and family reputation. This pattern is consistent with Goffman’s [45] theory of stigma, which suggests that when a discrediting attribute is not immediately visible, individuals may engage in “information management” to avoid disclosure and protect their social identity [56]. Prior research similarly shows that concealment can function as a coping strategy through which women manage stigma, avoid social sanctions, and preserve moral respectability within the family and wider community [11, 14]. By contrast, men’s smoking is more often practised openly in public spaces and does not require the same concealment, reflecting its wider social acceptance and its closer alignment with masculine role expectations [11, 17, 48]. Consistently, some male participants in our study stigmatised women who smoke while expressing little concern about their own smoking, highlighting a clear gendered double standard. A patriarchal reading suggests that this unequal need for concealment reflects broader gendered power relations: women bear the emotional and practical burden of managing their smoking identity because their behaviour is more closely monitored and judged than men’s.

Importantly, female participants in the present study not only recognised the stigma surrounding women’s smoking but also often rationalised it through sociocultural expectations. This differs from findings in more liberal contexts, where women may more openly contest or resist smoking-related stigma [37]. Gender role theory helps explain this pattern by showing that gender norms are not only imposed externally but are also learned and internalised through socialisation. As a result, women may come to endorse and reproduce expectations linked to modesty, respectability, and family reputation, even when these expectations restrict their own behaviour [39]. Patriarchal theory offers a complementary explanation: Kandiyoti’s [55] concept of the “patriarchal bargain” suggests that women may gain respectability, protection, and social standing by conforming to dominant gender norms. Within this arrangement, criticising other women’s behaviour can become a way of demonstrating one’s own moral worth and distancing oneself from a stigmatised identity. This helps explain why some women in our study reproduced the double standard surrounding smoking, even when they recognised its unfairness. Repeated exposure to normative messages such as “good women do not smoke” may further internalise these beliefs and lead women to project them onto others, thereby reinforcing gendered double standards [11, 14, 33, 54, 57].

Overall, our results suggest that, beyond the general health and social-psychological consequences of smoking, Saudi women may face intense blame and heightened fears of damaging family honour or reducing marriage prospects. These pressures may increase stress, guilt, and social isolation, potentially reinforcing smoking as a maladaptive coping strategy rather than reducing it [11, 14, 34]. Although excessive stigma may sometimes discourage women from experimenting with smoking, existing evidence suggests that its broader effects are more harmful than protective. Stigma can undermine cessation by discouraging women from seeking social support or professional help and by reducing their engagement with evidence-based quitting interventions. It can also encourage concealment, contributing to underreporting of smoking status, delayed diagnosis of smoking-related illnesses, and wider gender-based health disparities [11, 18, 22, 33, 34]. Thus, rather than promoting cessation, excessive stigma may push women further into secrecy, limiting open discussion, reducing healthcare engagement, and weakening the effectiveness of tobacco control policies.

From a theoretical standpoint, these dynamics show the value of using the four complementary perspectives adopted in this study. Gender role theory explains why women’s smoking is treated as a violation of accepted femininity, as it conflicts with expectations of modesty, restraint, and family responsibility. Patriarchal theory helps explain why this judgment is so unequal by showing how women’s behaviour is often more closely monitored and more strongly linked to family reputation and social respectability. Social control theory highlights the regulatory function of stigma, showing how families and communities may use disapproval, blame, and shame to discourage behaviours seen as threatening to social order or shared moral values. Finally, Goffman’s stigma theory captures the lived consequences of this process, including concealment, fear of disclosure, damaged identity, and reluctance to seek support. Taken together, and understood within the specific honour-based, religious, and cultural dynamics of the Saudi context, these perspectives offer a more complete account of how gendered smoking stigma operates socially and psychologically. They also point to the need for gender-sensitive and culturally informed tobacco control interventions that address not only smoking behaviour itself, but also the gendered double standards and social pressures that shape women’s experiences of smoking.

### Implications

Addressing smoking among Saudi women requires moving away from stigma-based and moralising approaches toward gender-sensitive tobacco control strategies. Public health messages should frame smoking primarily as a health issue rather than a moral failing, emphasising cessation support, stress management, and well-being instead of reinforcing stereotypes of women as irresponsible mothers, wives, or daughters. Involving women, including former smokers, in the design of prevention and cessation campaigns may also help ensure that messages reflect lived experiences and avoid unintended shame. At the clinical level, confidential and women-sensitive cessation services, such as anonymous hotlines, mobile applications, women-only clinics, and private primary-care consultations, may reduce barriers to help-seeking. Healthcare providers should also receive training to avoid blaming language, protect confidentiality, and provide non-judgmental support, particularly in contexts related to pregnancy and reproductive health.

Beyond individual-level interventions, schools, universities, and community programs could help foster critical awareness of gendered double standards in smoking-related stigma. Such initiatives may encourage both women and men to reflect on why identical behaviours are judged differently by gender and how these judgments contribute to stigma, secrecy, and unequal health outcomes. Collaboration with respected religious and community leaders may further strengthen these efforts by promoting anti-smoking messages for all individuals while also discouraging unjust shaming, gossip, and moral surveillance of women. More broadly, tobacco control efforts should be situated within wider initiatives that support women’s autonomy, mental health, and social participation, as this may reduce reliance on smoking as a coping strategy under conditions of stress and social pressure. These implications are broadly consistent with previous studies conducted in Saudi Arabia (e.g., [32–34, 58].

### Limitations and future research

The present study has several limitations. First, although the sample is appropriate for qualitative research seeking depth and diversity of views, the findings cannot be generalised to the wider Saudi population. Views on smoking and gender may differ across regions, social classes, educational backgrounds, and local cultural contexts, particularly given the social diversity within Saudi Arabia. Second, although the sample included women and men, as well as smokers and non-smokers, the number of current smokers, especially women who smoke, was relatively small. Consequently, the findings reflect perceptions of women’s smoking more than the lived experiences of women smokers themselves.

Future research could address these limitations by using larger and more diverse samples that include participants from different regions, age groups, social classes, marital statuses, and educational backgrounds. Quantitative surveys based on the themes identified in this study could examine how widespread specific forms of stigma are and how they relate to factors such as religiosity, gender ideology, and personal smoking status. Further qualitative work should also focus more directly on women who smoke, using in-depth interviews to explore how they negotiate stigma in their families, workplaces, healthcare encounters, and social networks. Comparative studies between smokers and non-smokers, or across generations, could clarify how social change in Saudi Arabia is reshaping attitudes toward smoking and gender. Finally, regional or cross-cultural comparisons, particularly across Arab or Muslim societies, could help distinguish context-specific features of smoking-related stigma from patterns that are more widely shared.

## Conclusion

This study shows that smoking-related stigma in Saudi Arabia cannot be understood as a response to smoking alone; rather, it is deeply shaped by gendered expectations about femininity, morality, family honour, and social respectability. While men’s smoking was largely treated as an unhealthy but ordinary behaviour, women’s smoking was constructed as a broader social and moral transgression, with consequences extending beyond the individual woman to her family and social identity. By bringing together gender role theory, patriarchal theory, social control theory, and stigma theory, the study offers a more nuanced understanding of how gendered smoking stigma is produced, justified, internalised, and managed in everyday life. The findings, therefore, point to the need for tobacco control approaches that reduce smoking without intensifying shame, secrecy, or gendered blame. Effective interventions should address smoking as a health issue while also challenging the social conditions that make women’s smoking uniquely stigmatised.

## Supplementary Information

Supplementary Material 1.

## Data Availability

The datasets generated and/or analysed during the current study are not publicly available due to ethical restrictions and concerns regarding participant privacy.
