# Supplementary material for: Stigma toward female smokers: gendered perceptions and moral judgment
Source: BMC Psychol. 2026 Jun 9;14:1254. doi: 10.1186/s40359-026-04946-z (PMC13504815; doi:10.1186/s40359-026-04946-z)
Supplement: Supplementary file 1 [file 40359_2026_4946_MOESM1_ESM.docx]

**Interview Questions**

- When you hear the phrase “a woman who smokes,” what is the first thing that comes to mind?
- How is a woman who smokes usually perceived in our society?
- How is a man who smokes usually perceived in our society?
- What do you think shapes these perceptions of women who smoke (e.g., family, peers, media, cultural or religious norms)?
- Do you think people judge men’s and women’s smoking differently? Why?
- In your view, how might smoking affect a woman’s life (e.g., work, education, marriage, social relationships)?
- And how might smoking affect a man’s life in these areas?
- Do you think smoking influences how a woman’s femininity is perceived? How?
- Do you think smoking influences how a man’s masculinity is perceived? How?
- Do you think recent social changes and expanded opportunities for women (e.g., travel, work) relate in any way to women’s smoking? How?
- Do you think these societal perceptions might change over time? How?
